# Supplementary material for: Imparting multi-functionality to covalent organic framework nanoparticles by the dual-ligand assistant encapsulation strategy
Source: Nat Commun. 2021 Jul 27;12:4556. doi: 10.1038/s41467-021-24838-7 (PMC8316466; doi:10.1038/s41467-021-24838-7)
Supplement: Supplementary file 1 — Supplementary information [file 41467_2021_24838_MOESM1_ESM.pdf]

Supplementary information

Imparting multi-functionality to covalent organic framework nanoparticles by the dual-ligands assistant encapsulation strategy

Chen et al.

Materials

1-octadecene (ODE, 90 %), oleic acid (OA, 90 %) Gadolinium (III) chloride anhydrous ($GdCl_3$, 99.99 %), yttrium (III) chloride anhydrous (YCl_3, 99.9 %), ytterbium (III) chloride anhydrous ($YbCl_3$, 99.9 %), erbium (III) chloride anhydrous ($ErCl_3$, 99.9 %), neodymium (III) chloride hexahydrate ($NdCl_3$, 99.9 %) and sodium trifluoroacetate (Na-TFA, 98 %) were purchased from Sigma-Aldrich Trading Co., Ltd (Shanghai, China). Tetraethyl orthosilicate (TEOS), polyethylenimine (PEI, Mw ~10000), 5,10,15,20-Tetrakis(4-aminophenyl)porphyrin (TAPP), Polyvinylpyrrolidone (PVP, Mw ~58000), cis-Diammineplatinum dichloride (cisplatin) and 1, 3-Diphenylisobenzofuran (DPBF) were bought from Aladdin Chemistry, Co., Ltd. 1,3,5-tris(4-aminophenyl)benzene (TAPB), 2,5-dimethoxyterephthalaldehyde (DMTP), Triformylbenzene, 2,5-Dihydroxy-1,4-benzenedicarboxaldehyde and p-phenylenediamine were provided by Energy Chemical Co., Ltd. Fetal bovine serum (FBS), penicillin-streptomycin, trypsin, and Roswell Park Memorial Institute (RPMI) 1640 medium were provided from Gibco Life Technologies Co. ROS Assay Kit (DCFH-DA) were obtained from Beyotime Biotechnology (Shanghai, China). Paraformaldehyde was purchased from Beijing Dingguo Changsheng Biotechnology Co., Ltd. 4′,6-Diamidino-2-phenylindole (DAPI) was purchased from Bestbio. All other regents were of analytical grade and commercially available. Deionized (DI) water was used throughout the experiments.

Synthesis of seeds

SiO_2 nanoparticles: Typically, 20.0 mL of ethanol, 20.0 mL of methanol, 5.2 mL of water and 4.7 mL of ammonium hydroxide were mixed and heated to 55 °C for 15 min. Then 1.9 mL of TEOS were added into the solution slowly. The mixture was further stirred at 600 rpm for 2 h. The products were isolated by high-speed centrifugation and washed several times with ethanol.

Mesoporous silica nanoparticles (MSNs): Typically, 3.00 g of CTAB and 150 μL of TEA were dissolved in 60.0 mL of water and heated to 60 °C for 1 h. Then 16.0 mL of cyclohexane and 4.0 mL of TEOS were mixed and slowly added onto the upper layer of water phase. The mixture was further stirred at 200 rpm for 24 h. The products were isolated by high-speed centrifugation and washed several times with ethanol. To remove the surfactant of CTAB, the MSNs was dispersed in HCl-ethanol solution (10.0 wt%) and refluxed for 12 h, the process was repeated several times.

Other seeds: The Fe_3O_4 nanospheres, gold nanoshell, CuS nanoplates, and Fe_2O_3 ellipsoids were

synthesized by the reported method in previous publications. [1-4]

Synthesis of $NaYF_4$:Yb/Er@$NaGdF_4$ UCNPs

Synthesis of $NaYF_4$:Yb/Er nanocrystals: Hexagonal phase $NaYF_4$:20%Yb/2%Er nanocrystals were synthesized according to a previously reported thermolysis method.[5] Typically, YCl_3 (0.78 mmol), $YbCl_3$ (0.20 mmol), $ErCl_3$ (0.02 mmol), OA (6.0 mL) and ODE (15.0 mL) were mixed together and heated to 140 °C under vacuum until a clear solution formed. After that, the solution was cooled down to room temperature. Then, a methanol solution (10.0 mL) of ammonium fluoride (4.00 mmol) and sodium hydroxide (2.50 mmol) was added and stirred for 1 h at 50 °C. The mixture was then heated to 70 °C and maintained under vacuum for half an hour to remove the methanol. Afterward, the solution was heated to 270 °C (~ 10 °C/min) and maintained for 90 min under a gentle argon flow. Then, the solution was cool down to room temperature and the nanocrystals were centrifuged and washed twice with ethanol. The nanocrystals were finally dispersed in 10.0 mL of cyclohexane for further use.

Gd-OA (0.10 M) shell precursor: 2.50 mmol of $GdCl_3$, 10.0 mL of OA, and 15.0 mL of ODE were mixed in a round-bottom flask and heated at 140 °C under vacuum with magnetic stirring for 30 min to remove residual water and oxygen. Then the colorless Gd-OA precursor solution (0.10 M) was obtained.

Na-TFA-OA (0.40 M) shell precursor: A mixture of Na-TFA (4.00 mmol) and OA (10.0 mL) was loaded in a container at room temperature under vacuum with magnetic stirring to remove residual water and oxygen. Then the colorless Na-TFA-OA precursor solution (0.40 M) was obtained.

Synthesis of core@shell structured nanocrystals: The core@shell nanoparticles were fabricated by using the one-pot successive layer-by-layer (SLBL) protocol, which was developed by our group.[6] 2.5 mL of the purified $NaYF_4$:Yb/Er core nanoparticles solution (~ 0.25 mmol) were mixed with 4.0 mL of OA and 6.0 mL of ODE. The flask was pumped down at 70 °C for 30 min to remove cyclohexane. After that, the system was switched to Ar flow and the reaction mixture was further heated to 280 °C at a rate of ~ 20 °C/min. Then pairs of Gd-OA (0.10 M, 1.0 mL) and Na-TFA-OA (0.40 M, 0.50 mL) precursors were alternately introduced by dropwise addition at 280 °C and the time interval between each injection was 15 min. Finally, the obtained

$NaYF_4$:Yb/Er@$NaGdF_4$ core@shell nanoparticles were precipitated and washed in the same way as the core nanoparticles, and dispersed in cyclohexane.

The core-shell $NaGdF_4$:5%Nd@$NaGdF_4$ nanoparticles were also synthesized by the similar protocols described above except that the precursors were correspondingly replaced.

Surface modification with PEI

The as-prepared seeds (*e.g.* SiO_2 nanospheres) were dispersed in water at a concentration of 20 mg/mL, then 1.0 mL of PEI aqueous solution (100 mg/mL) was slowly added into the dispersion. The mixture was stirred at room temperature for 1 h. Then, the PEI modified nanoparticles were collected and washed by high-speed centrifugation.

Selectively etching of SiO_2 from the core@shell structured SiO_2@COF

To obtain bowl-shape COF, the SiO_2@COF prepared with 2.0 mg of DMTP was dispersed in 2 M NaOH aqueous solution and stirred overnight. The products were obtained and repeatedly washed by centrifugation. Similarly, the SiO_2@COF prepared with 4.0 mg DMTP can transform to hollow structured COF after the etching process.

Synthesis of functional UCNPs@SiO_2

To synthesize UC-COF, the oleic acid-stabilized upconversion $NaYF_4$:Yb/Er@$NaGdF_4$ nanocrystals were transferred to hydrophilic by coating a silica shell. Inverse microemulsion method was used to synthesize $NaYF_4$:Yb/Er@$NaGdF_4$@SiO_2 according to our previous report.[7] Then the $NaYF_4$:Yb/Er@$NaGdF_4$@SiO_2 nanoparticles were modified with PEI as described above.

Synthesis of SiO_2@LZU-1 and porphyrin-based COF coated SiO_2

For the synthesis of SiO_2@LZU-1, 6 mg of PEI modified SiO_2 nanospheres and 40.0 mg of PVP were added into 10.0 mL mixed solution of *o*-dichlorobenzene/n-butyl alcohol (v/v = 1:1). The dispersion was treated with ultrasonic for more than 0.5 h. Then, 3.5 mg of triformylbenzene and 3.5 mg of p-phenylenediamine were added. After stirring for 5 min, 100 μL of glacial acetic acid was added. The reaction was maintained at room temperature for 4 h. Afterward, 400 μL of acetic acid and 480 μL of DI water was added. The mixture was degassed by three freeze-pump-thaw cycles, sealed under vacuum, and kept at 120 °C for 5 days. The products were obtained and washed with acetone by centrifugation.

For the growth of porphyrin-based COF, the PEI modified SiO_2 nanospheres were used as core

and the synthetic procedures were identical with the synthesis of UC-COF.

Characterization

The morphology of prepared nanoparticles was observed by transmission electron microscopy (TEM) under accelerated voltage of 200 kV (FEI, American). Scanning electron microscopy (SEM) images were captured using field emission scanning electron microscopy (FESEM, Hitachi S-4800, Japan). X-ray diffractions (XRD) of samples were recorded on a D/max-2550 PC X-ray diffractometer (XRD; Rigaku, Japan). UV–vis–NIR absorption spectra were measured on a Shimadz spectrophotometer (UV-3150) (Japan). Size distribution and Zeta potential of the samples were recorded by using Zetasizer Nano ZS apparatus (Malvern, UK). The concentrations of Gd element were analyzed by a Leeman Prodigy inductively coupled plasma-atomic emission spectroscopy (ICP-AES) system (Hudson, NH03051, USA). Fourier transform infrared (FTIR) spectra were recorded using a NICOLET MX-1E FTIR spectrometer.

In vitro cytotoxicity evaluation

The cells were cultured in standard Roswell Park Memorial Institute (RPMI) 1640 medium supplemented with 10% (v/v) FBS, 100 mg mL^{-1} streptomycin and 100 U mL^{-1} penicillin at 37 °C in a humidified incubator with 5% CO_2.

The cytotoxicity of the prepared UC-COF was assessed using standard CCK-8 assay. 4T1 or HUVECs were seeded in 96-well plates (10^4 cells/well) for 24 h. After that, the cells were incubated with fresh medium containing different concentrations of UC-COF (400, 200, 100, 50, 25, 12.5, 6.25 µg/mL) for another 24 h. Then, the medium was discarded and the cells were slightly washed by PBS, followed by the standard CCK-8 protocol according to the manufacturer's instruments. Cells treated with pure medium without UC-COF was used as control group. Four parallel experiments were performed for each group.

In Vitro Cell Uptake

The cell uptake of UC-COF was first investigated by confocal laser scanning microscopy (CLSM, Olympus FV900). 4T1 cells were seeded in glass-bottom culture dish (3.0×10^5 cells per dish) and incubated with UC-COF at two different pH conditions (7.4 and 6.5) for 4 h. Subsequently, the cells were slightly rinsed with PBS, fixed with 4% paraformaldehyde, and stained with DAPI for direct observation.

In vitro ROS detection

To evaluate the ROS generation ability, 200 μL of UC-COF in DMSO was mixed with 10 μL of DPBF solution (6 mM). The mixed solution was then irradiated by 980 nm laser (1 W/cm^2). The change of absorption of DPBF at 420 nm was recorded by a UV-vis spectrophotometer.

Tissue depth-dependent PDT

Further, the impact of tissue depth on killing effect of NIR light-triggered and visible light-triggered PDT was also investigated. The cells were subjected to the same treatment as described above, except that the cell plates were placed under a cylindrical culture dish with 4-mm thickness 1% Intralipid®, which was applied as a simulated tissue due to its similar scattering characteristics.[8] The cell viability of different groups was evaluated by CCK-8 assay, and the cells were stained with Calcein-AM/PI after different treatments for CLSM observation.

Statistical analysis

At least three independent groups were performed throughout experiment. Significant difference between different groups was analyzed by one-way analysis of variance (ANOVA) and Turkey's post hoc test. The criterion was expressed as *$P < 0.05$ and **$P < 0.01$.

Supplementary Figures

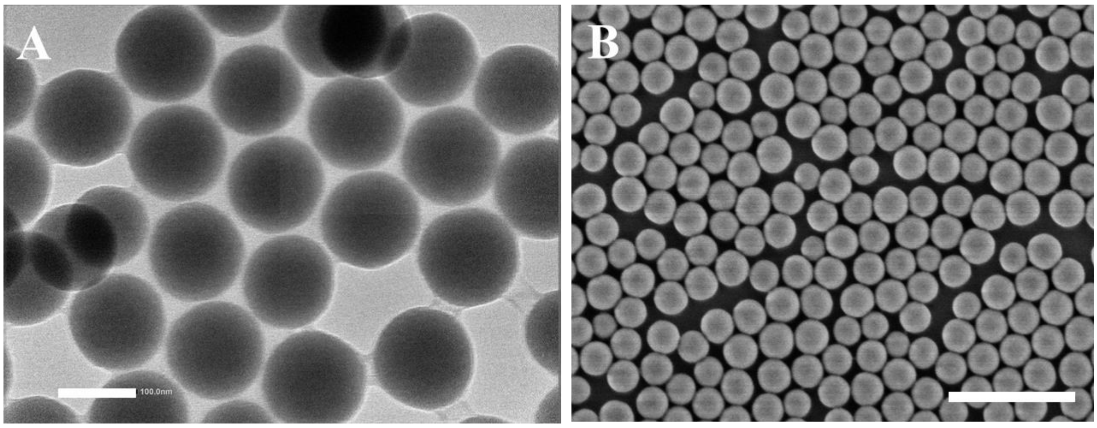

Supplementary Figure 1. TEM and SEM images of PEI modified SiO_2 nanoparticles. Scale bars are 100 nm in A and 500 nm in B. A representative image of three replicates is shown. SiO_2 nanoparticles are still very uniform after the surface modification with the PEI. The diameter is about 120 nm.

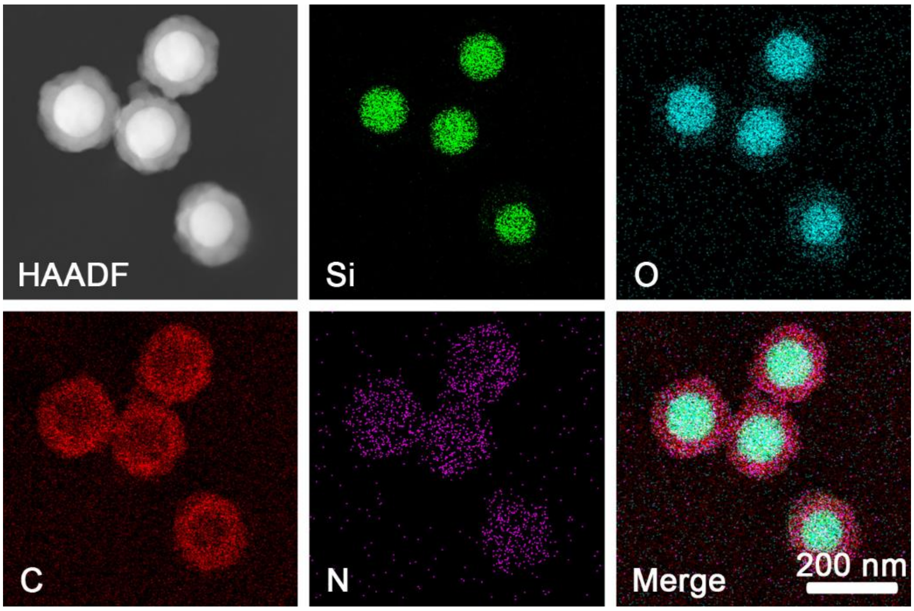

Supplementary Figure 2. Element mappings of the SiO_2@COF nanoparticles. A representative image of three replicates is shown. The core@shell structure can be clearly observed. The Si and O elements were mainly located in the center, while the C and N elements from COF were distributed in the outer layer.

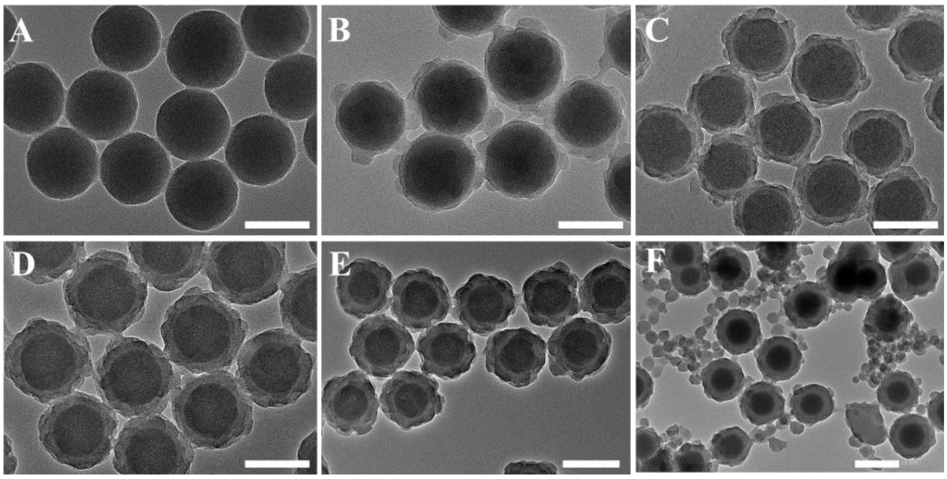

Supplementary Figure 3. TEM images of core@shell structured SiO_2@COF nanoparticles with different shell thickness synthesized by using different amount of DMTP monomer (1.5 equivalent of TAPB): (A) 0.5 mg, (B) 1 mg, (C) 2 mg, (D) 4 mg, (E) 8 mg and (F) 16 mg. Scale bar are 100 nm in A-D, and 200 nm in E and F. A representative image of three replicates is shown. The thickness of the COF shell can be well tuned. With the increasing of amount of monomers, the shell thickness of resultant SiO_2@COF nanoparticles is accordingly increased. However, excessive amount of monomers would give rise to the phase separation of COF nanoparticles in solution.

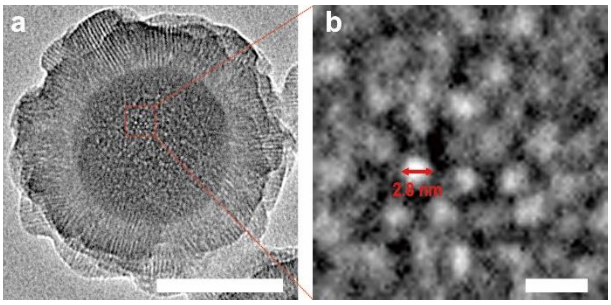

Supplementary Figure 4. (a, b) HRTEM image of single SiO_2@COF nanoparticle. Scale bars are 100 nm in (a) and 5 nm in (b). A representative image of three replicates is shown. The fringes belong to the vertical pores perpendicular to the 2D plane of the COF layers. The projected view of the ~ 2.8 nm pores can also be clearly observed. So, we can conclude that the 2D plane of the COF layers is parallel to the SiO_2 surface, and the fringes can be assigned to the periodic pores perpendicular to the 2D plane of the COF layers and the SiO_2 surface.

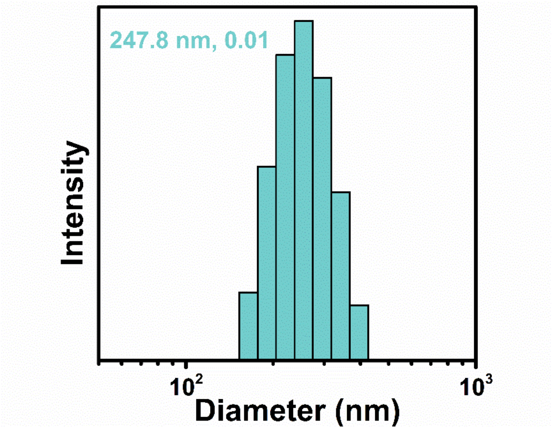

Supplementary Figure 5. Dynamic laser scattering (DLS) of the synthesized SiO_2@COF nanoparticles. The size distribution of SiO_2@COF nanoparticles is very narrow, and the polydispersity is 0.01, demonstrating the monodispersity of obtained SiO_2@COF.

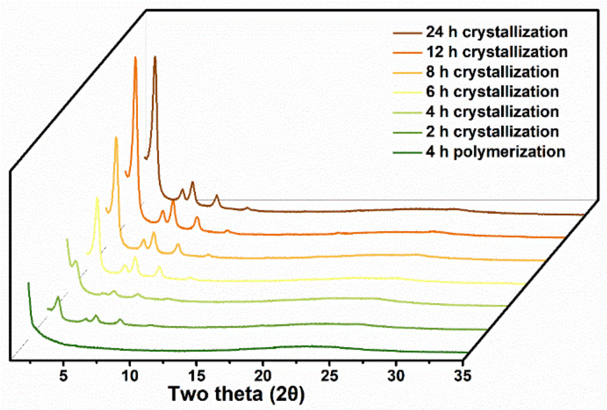

Supplementary Figure 6. XRD patterns of the SiO_2@COF nanoparticles after thermal treatment for different time. No peaks can be detected after 4 h reaction at room temperature. The characteristic diffraction peaks start to appear after the thermal treatment. Moreover, the crystallization time also affect the crystalline degree of SiO_2@COF. It was noticed that the samples exhibit the strongest intensity of diffraction peaks after 12 h thermal treatment, and peak intensity slightly decreased after 24 h, which probably due to the reversible rearrangement during the crystallization of COF shell.

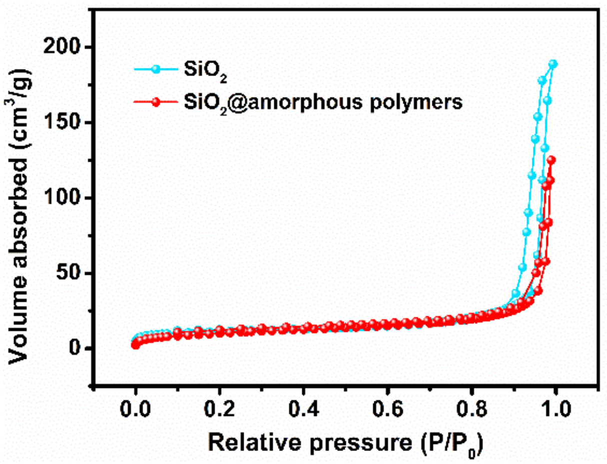

Supplementary Figure 7. Nitrogen absorption-desorption isotherm of bare SiO_2 and SiO_2@amorphous-polymers. The specific surface area of SiO_2@amorphous-polymers is lower than that of bare SiO_2, suggesting that the porosity of SiO_2@COF is attributed to the highly crystalline COF shell after thermal crystallization.

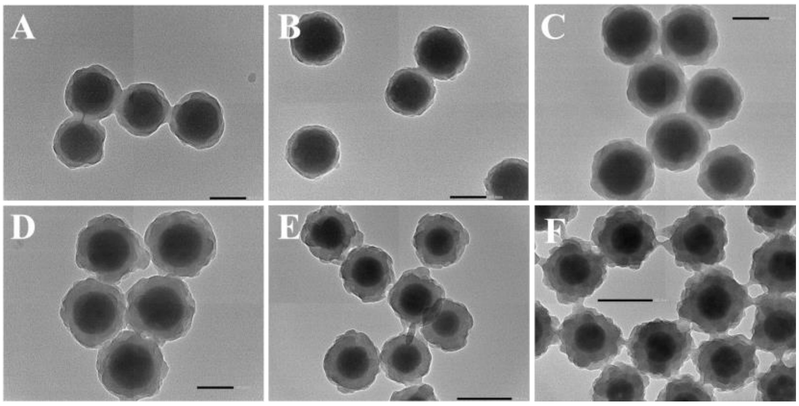

Supplementary Figure 8. TEM images of SiO_2@COF nanoparticles obtained at different reaction time: (A) 4 h after room temperature polymerization, and thermal treatment for (B) 2 h, (C) 4 h, (D) 8 h, (E) 12 h and (F) 24 h. Scale bar is 100 nm in A-D, and 200 nm in E and F. A representative image of three replicates from each group is shown. The polymer shell is successfully formed after 4 h under room temperature. In addition, the COF shell become thicker after the thermal crystallization, indicating that the crystallization process is accompanied with the reconstruction of the polymer shell to form the highly crystalline COF shell.

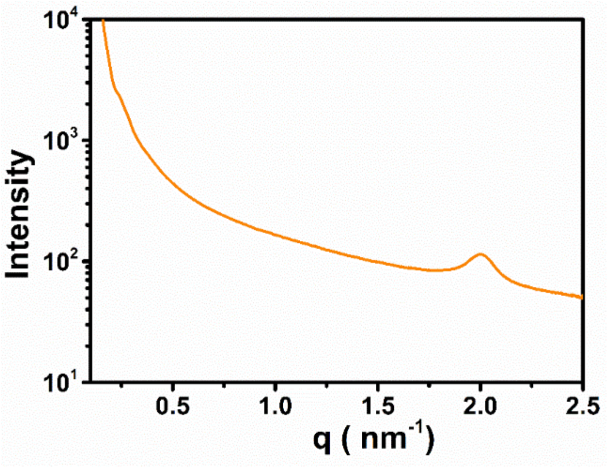

Supplementary Figure 9. Small angle X-ray scattering (SAXS) of prepared SiO_2@COF nanoparticles. The peak at 2.0 nm^{-1} corresponds to (100) facet of DMTP-TAPB-COF, implying the high crystallinity of COF shell.

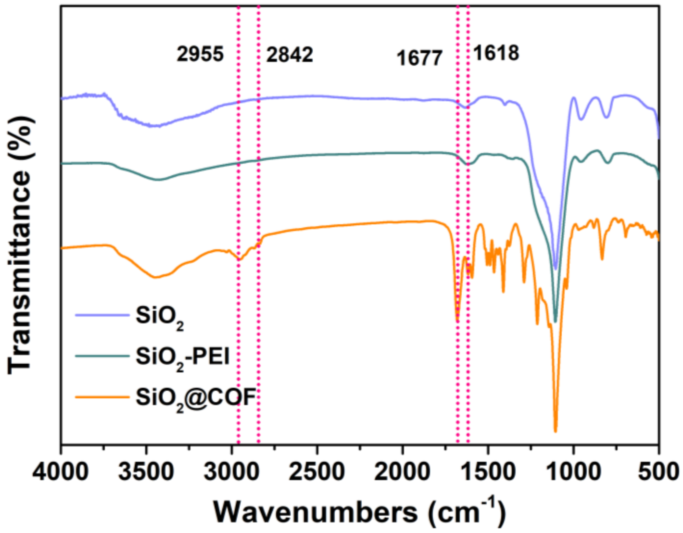

Supplementary Figure 10. FTIR spectra of prepared samples. In comparison to the bare SiO_2 and PEI modified SiO_2, the synthesized SiO_2@COF displays extra peaks at 1677 and 1618 cm^{-1}, which can be assigned to the −CHO groups and C=N stretching vibration in the COF shell, respectively. The peaks at 2955 and 2842 cm^{-1} are associated with the C−H vibration of $-CH_3$ groups in DMTP.

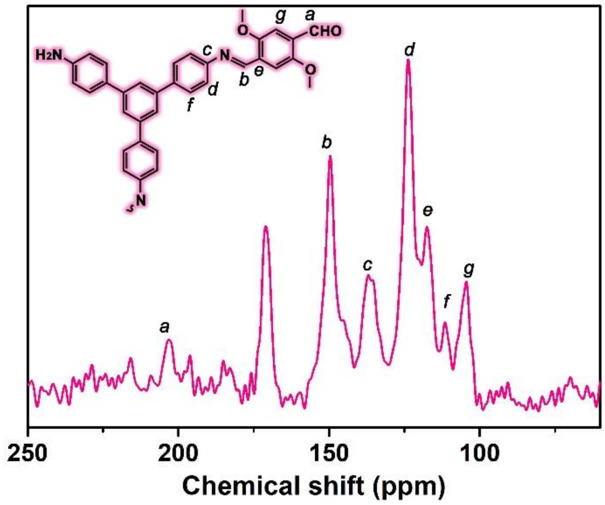

Supplementary Figure 11. Solid-state ^{13}C NMR spectrum of prepared SiO_2@COF. The characteristic peaks at 104, 111, 117, 123, 137, 150, and 203 ppm is assigned to the aromatic carbon of COF units, suggesting the formation of imine-based COF on SiO_2. The peak at 171 ppm can be attributed to the carbonyl signal of PVP.

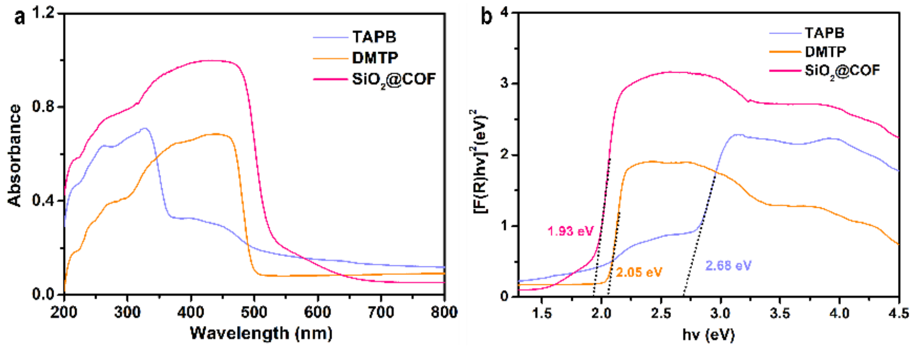

Supplementary Figure 12. (a) UV-vis reflectance spectra and (b) the band gap of different samples estimated from the UV-vis reflectance spectra. The results show that the SiO_2@TAPB-DMTP COF nanocomposites exhibit smaller band gaps compared with the corresponding molecular building blocks, demonstrating the cooperation between chromophores across the entire COF crystals.

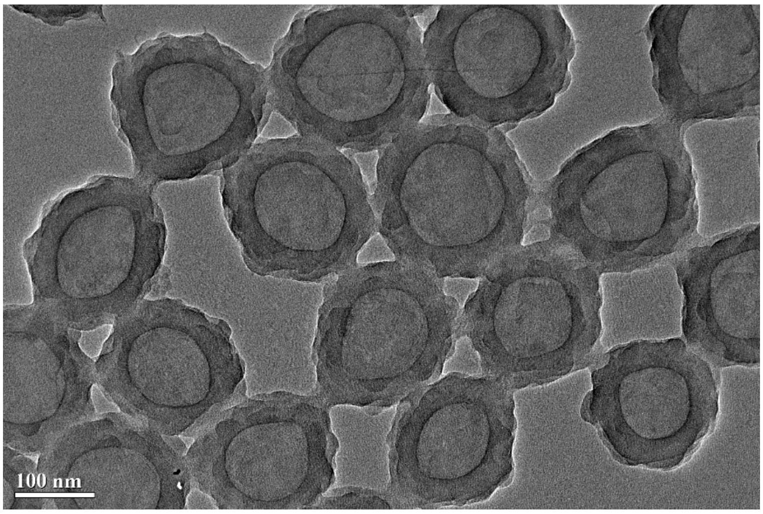

Supplementary Figure 13. TEM image of the hollow COF nanoparticles after the selective etching the SiO_2 core from SiO_2@COF nanoparticles. A representative image of three replicates is shown. Although there are some deformations for the cavity after removing the SiO_2 core, the hollow structure of COF is maintained.

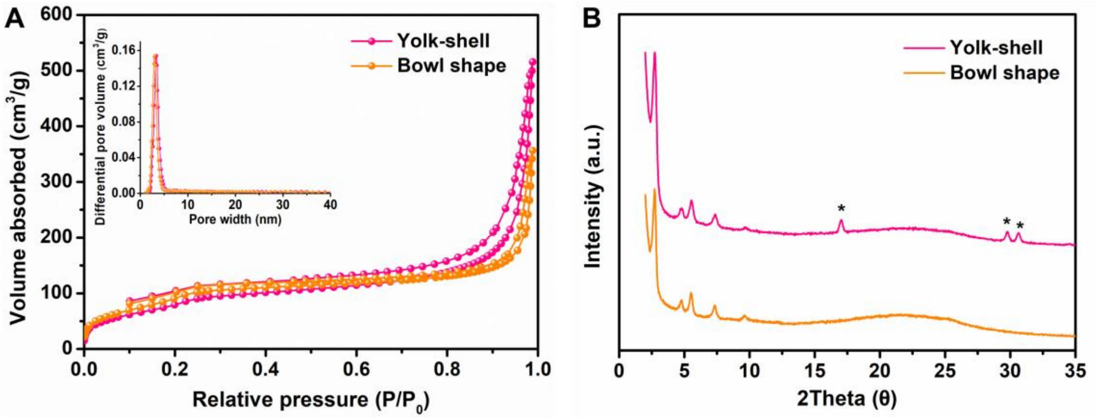

Supplementary Figure 14. (A) N_2 adsorption-desorption isotherms and pore distribution (inset image), and (B) XRD patterns of the synthesized bowl-shaped COF nanoparticles and yolk-shell structured UCNPs@COF in Figure 2d and 2f, respectively. The asterisk labeled peaks are assigned to the typical diffraction peaks of UCNPs. The results show that the porous structures of COF shell are not changed after the selective alkaline etching treatment, implying the high stability of prepared COF-based nanocomposites.

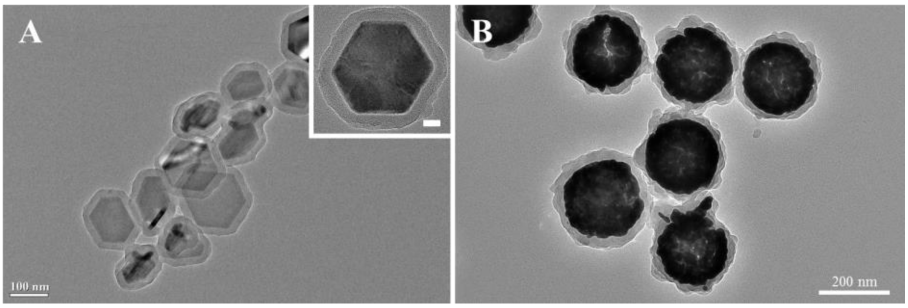

Supplementary Figure 15. TEM images of (A) CuS-nanoplate@COF and (B) Gold-nanoshell@COF nanocomposites. Inset in A is the high-resolution TEM image of CuS@COF, scale bar is 20 nm. A representative image of three replicates from each group is shown. The functional cores are uniformly encapsulated by the COF shell to form the core@shell structure, regardless of the composition, geometry or surface properties of the core.

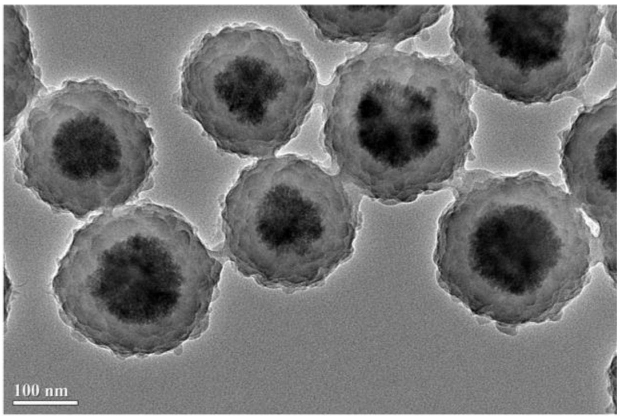

Supplementary Figure 16. TEM image of Fe_3O_4@COF nanocomposites. A representative image of three replicates is shown. The obtained core@shell structured Fe_3O_4@COF nanocomposites are very uniform.

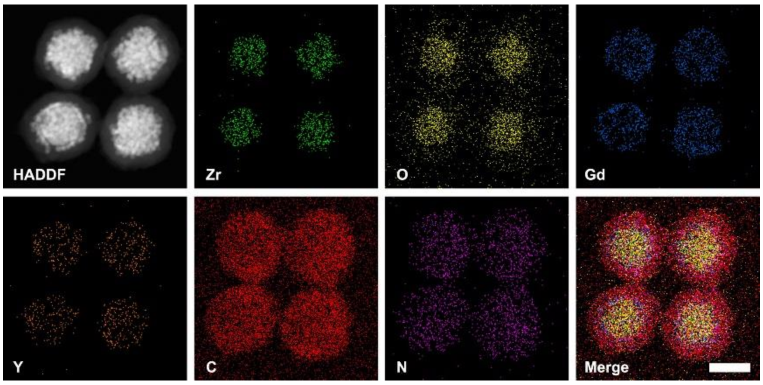

Supplementary Figure 17. The HAADF image and the element mapping of Zr-MOF@DCNPs@COF nanocomposites. A representative image of three replicates is shown. The expected elements were also detected and located in the correct position, suggesting the successful construction of core@satellites@shell structure of the nanocomposites.

A

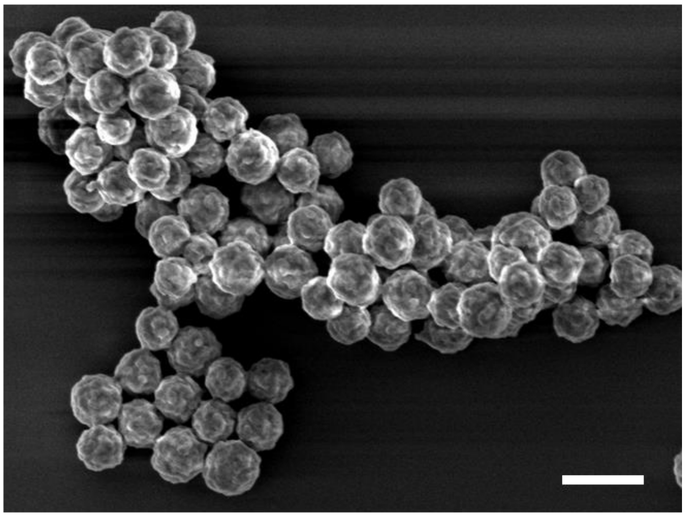

B

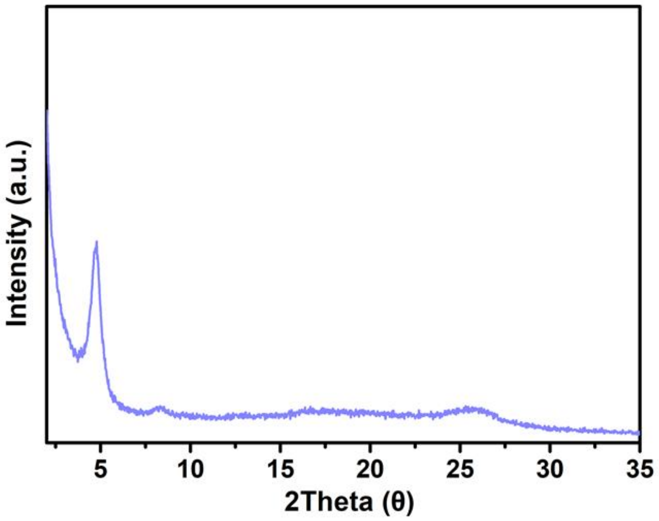

Supplementary Figure 18. (A) SEM image and (B) XRD pattern of prepared core@shell structured (c) SiO_2@LZU-1, scale bar is 400 nm. The obtained nanoparticles are uniform and monodisperse. The XRD pattern shows the typical diffraction peak at 4.7°, indicating the crystallization of LZU-1 COF shell.

A

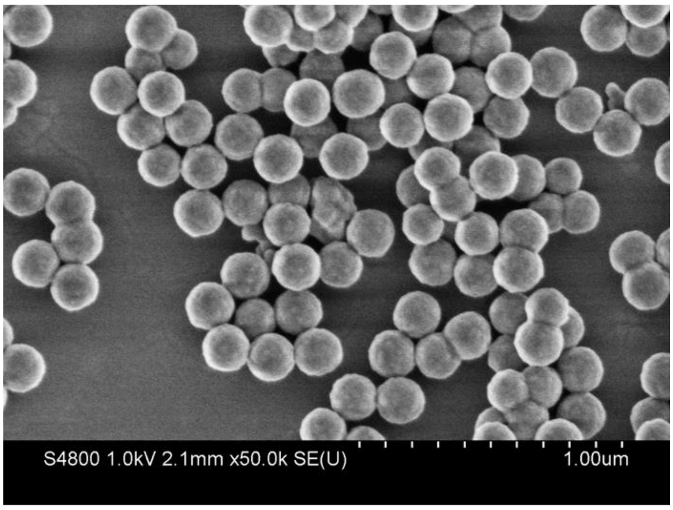

B

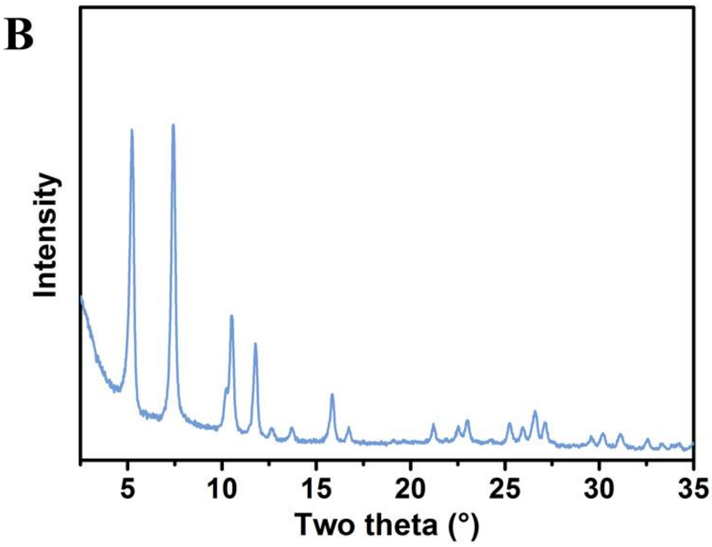

Supplementary Figure 19. (A) SEM image and (B) XRD pattern of prepared core@shell structured SiO_2@porphyrin-COF nanocomposites. The indicated location at 2.65°, 5.25°, 7.46°, 10.38° can be ascribed to the (110), (200), and (210), (220) facets of COF, respectively.

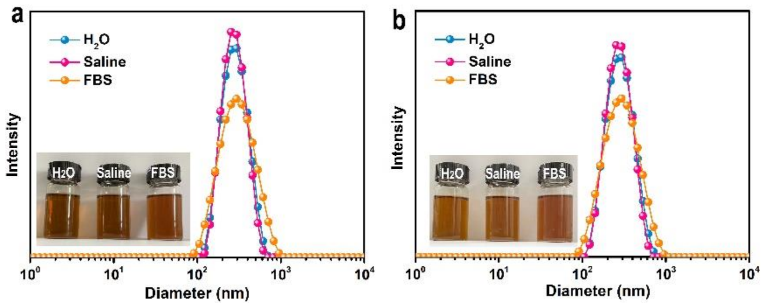

Supplementary Figure 20. Size distribution of UC-COF dispersed in different media for (a) 1 h and (b) 12 h. Inset represents the digital photos of the corresponding dispersions. No obvious agglomeration or precipitations was observed after 12 h incubation in different physiological conditions, suggesting the good colloidal stability of prepared UC-COF.

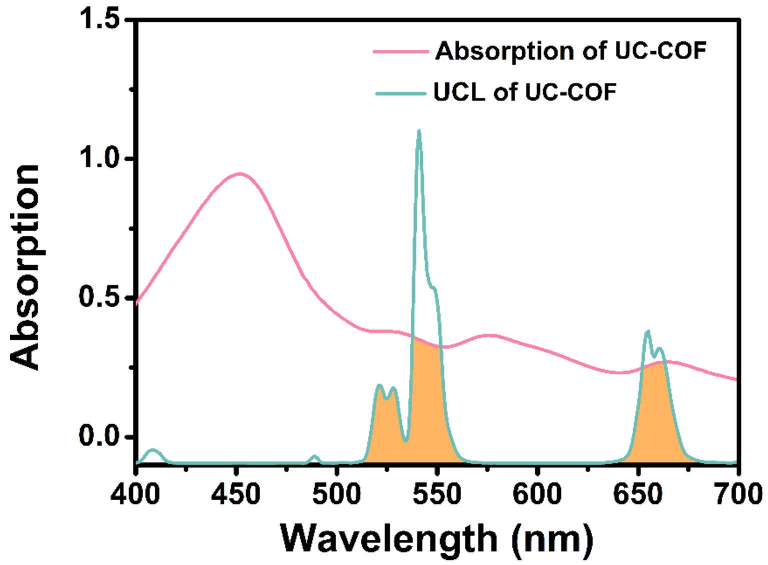

Supplementary Figure 21. The UV-vis spectrum (pink line) and upconversion emission spectrum of prepared UC-COF under excitation of 980 nm (green line). Upon irradiated by 980-nm NIR laser, the UC-COF can convert the NIR light to the visible light at ~ 545 and 655 nm, which is overlapped with the characteristic absorption peak of porphyrin.

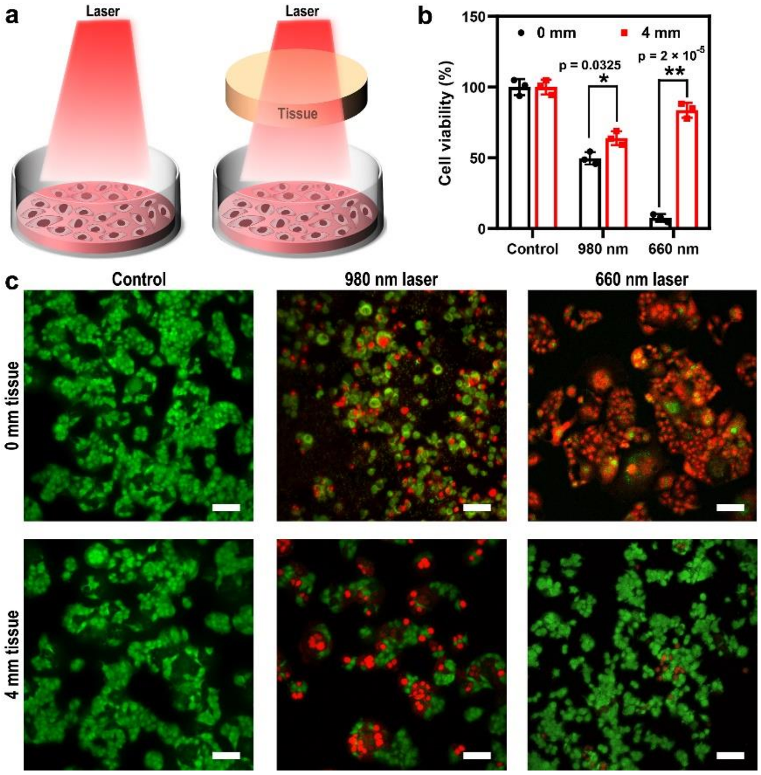

Supplementary Figure 22. (a) Schematic illustration of the designed experiment for PDT with or without the intervention of simulated tissue (1 % Intralipid). (b) Cell viability of 4T1 cells after treated with UC-COF under the irradiation of 980 or 660 nm laser, with or without the block of a 4 mm-thickness simulated tissue. Data are presented as mean ± s.d. derived from n= 3 biological independent samples. The statistical analysis was performed using one-way analysis of variance (ANOVA), followed by post hoc Tukey's method. Asterisks indicate a significant difference (*$p < 0.05$ and **$p < 0.01$). (c) The corresponding live-dead staining images of the treated cells in different groups, scale bars are 200 μm. A representative image of three replicates is shown. When the samples were directly irradiated by the laser, the photodynamic effect triggered by the 660-nm laser can kill more cancer cells than the NIR light-activated PDT. However, the killing efficiency decreases by ~75% for the 660 nm-triggered PDT when the light source was blocked by a 4 mm-thickness simulated tissue. In sharp contrast, for the NIR laser-triggered PDT, the killing efficiency only reduces by ~14%. The same tendency was also observed in the live-dead staining images. Even though the cancer cell killing efficiency of the UC-COF

group under NIR irradiation is lower than that of the mere COF group under visible light irradiation, the former one offers more favorable tumor treatment in deep tissue.

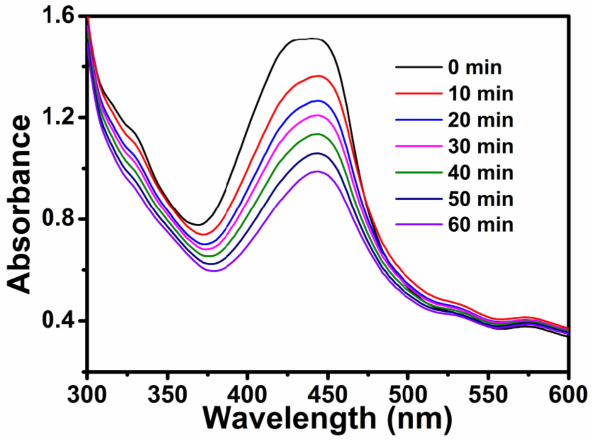

Supplementary Figure 23. The UV-vis spectra of DPBF solution mixed with UC-COF after irradiated by 980 nm laser for different time. The maximum absorbance (at 420 nm) of DPBF is clearly decreased upon exposure to 980-nm irradiation, indicating the NIR-activated ROS generation.

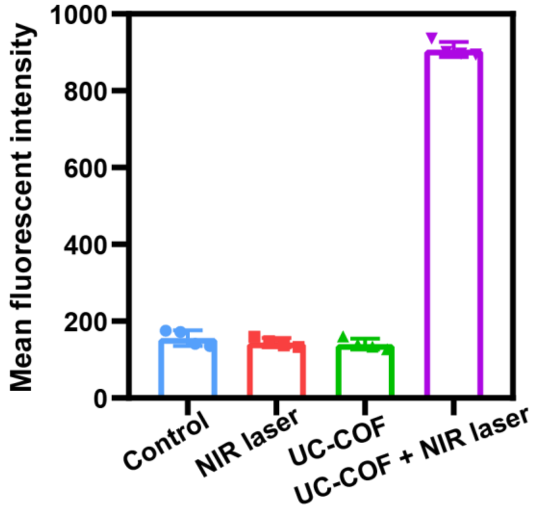

Supplementary Figure 24. Fluorescent intensity of the 2,7-dichlorodihydrofluorescein diacetate (DCFH-DA) ROS probe in 4T1 cells treated by different groups (the quantitative analysis of Figure 3c). Data are presented as mean ± s.d. derived from n= 4 biological independent samples. The strongest green fluorescence can be observed in the group of UC-COF + NIR laser, indicating the highest intracellular ROS level.

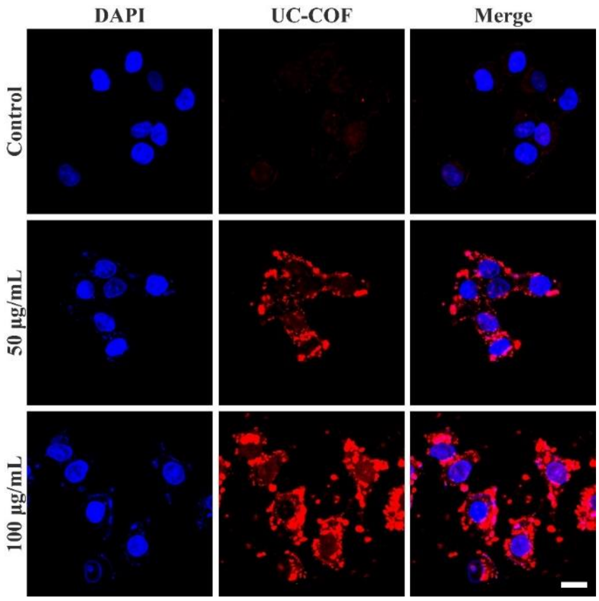

Supplementary Figure 25. CLSM images of 4T1 cells treated with UC-COF at different concentrations. Scale bar is 20 µm. The cell nuclei are surrounded by the red fluorescence from porphyrin of UC-COF, indicating the highly efficient endocytosis of the nanocomposites. A representative image of three replicates from each group is shown.

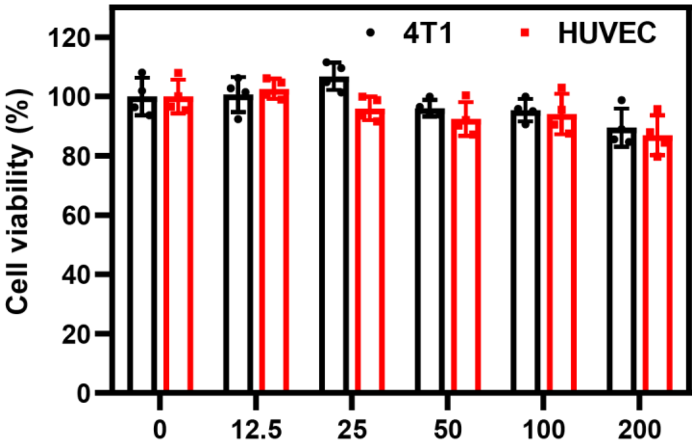

Supplementary Figure 26. Viability of 4T1 cells and HUVECs incubated with UC-COF for 24 h at different concentrations. Data are presented as mean ± s.d. derived from n= 4 biological independent samples. The results show that over 80 % cells are alive even exposed to a high concentration of 200 µg/mL, suggesting the low cytotoxicity of bare UC-COF.

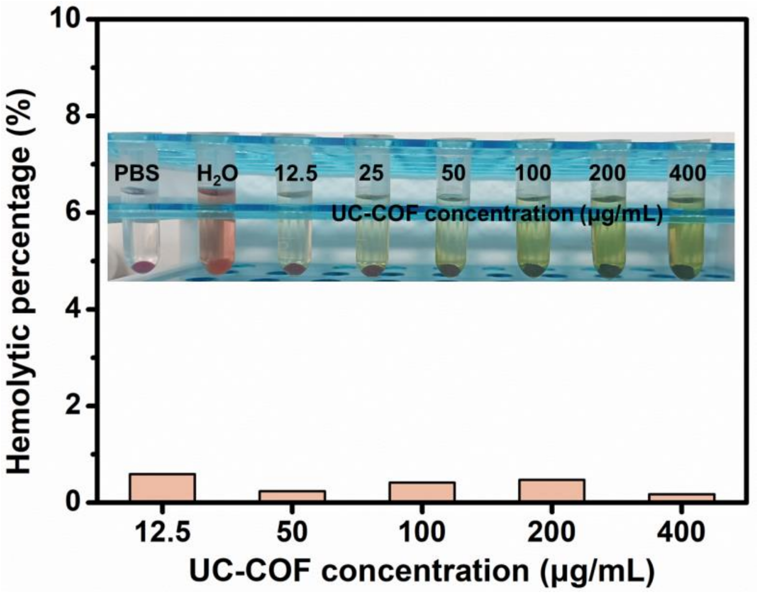

Supplementary Figure 27. The hemolysis percentage and photographs of red blood cells (RBCs) incubated with UC-COF for 24 h at different concentrations. All samples show negligible hemolytic effect even exposed to a high concentration of 400 μg/mL, suggesting the superior hemocompatibility of prepared UC-COF.

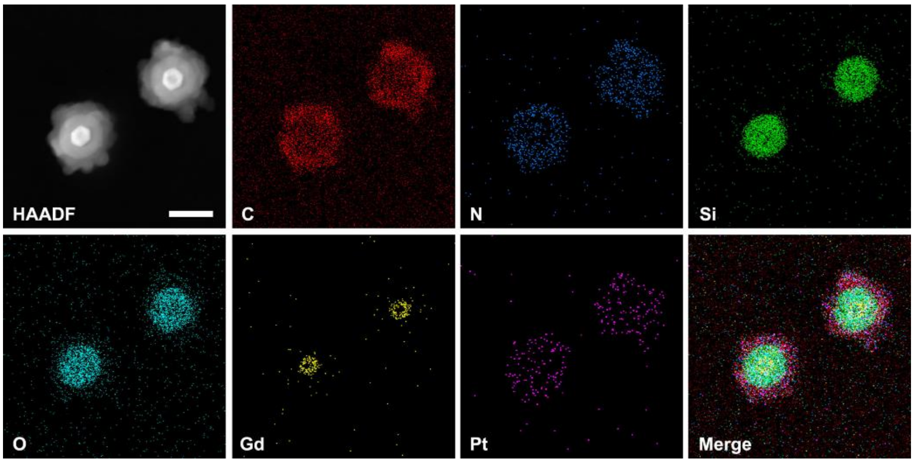

Supplementary Figure 28. The HAADF image and the element mapping results of cisplatin-loaded UC-COF. Scale bar is 100 nm. A representative image of three replicates is shown.

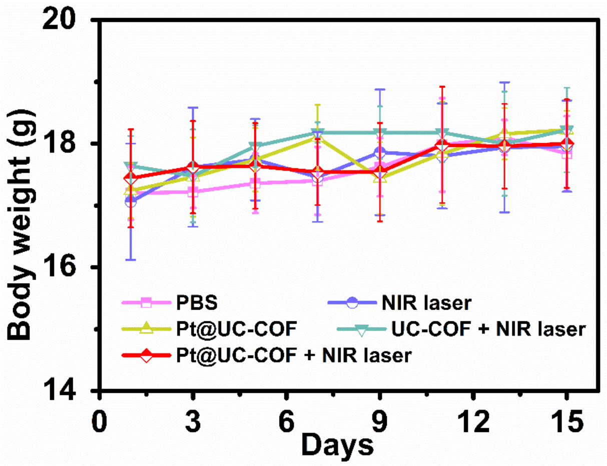

Supplementary Figure 29. The body weight of experimental mice during the treatment period. Data are presented as mean ± s.d. derived from n= 5 biological independent mice. No abnormal change of body weight is detected, which demonstrates that the implemented treatments are safe.

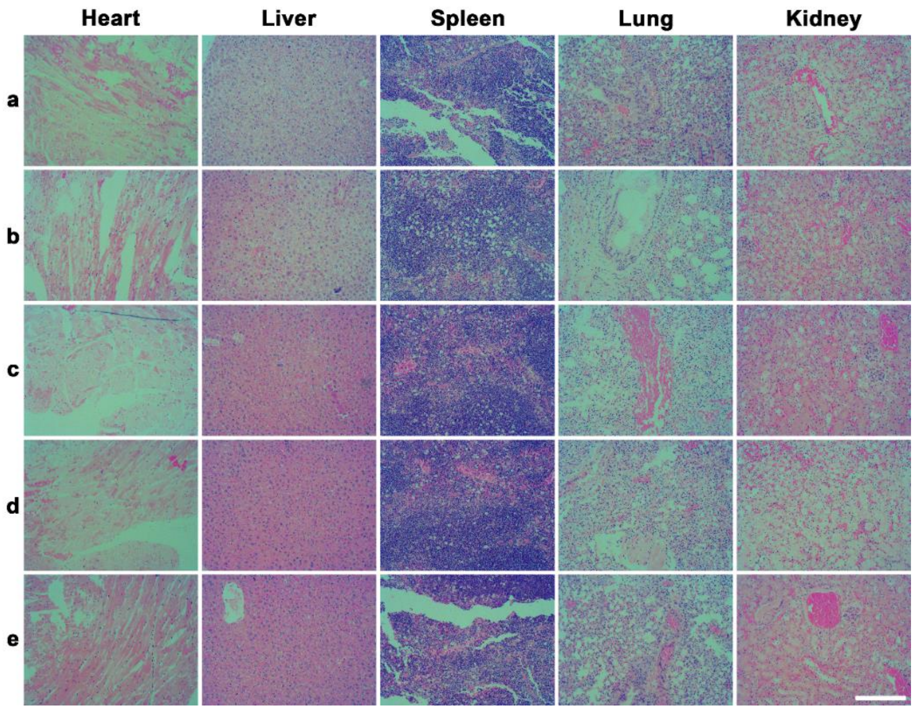

Supplementary Figure 30. The H&E-stained organ slice of mice after different treatments: (a) control, (b) NIR laser, (c) UC-COF-Pt, (d) UC-COF + NIR laser, and (e) UC-COF-Pt + NIR laser. Scale bar is 200 μm. A representative image of three replicates is shown. All samples exhibit no lesion or cell apoptosis after the treatments, indicating the safety of implemented treatments.

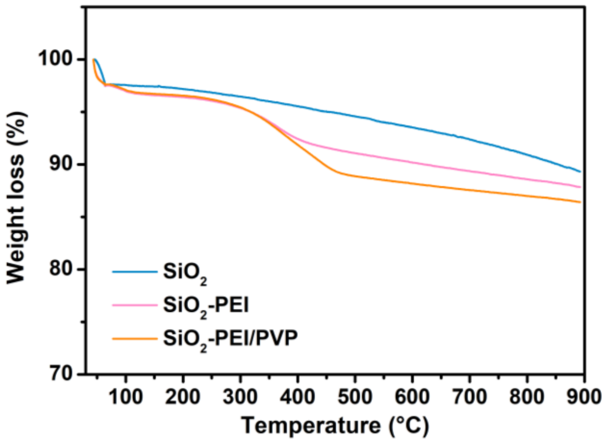

Supplementary Figure 31. TG curves of bare SiO_2, PEI modified SiO_2 and dual ligands modified SiO_2 nanospheres. The result shows that the weight loss of the samples was gradually increased in order from SiO_2 (10.6%), SiO_2-PEI (12.2%) to SiO_2-PEI/PVP (13.5%), suggesting the successive attachment of the dual ligands onto SiO_2 surface. The amount of the ligands on the SiO_2 is about 1.6 % and 1.3 % for PEI and PVP, respectively.

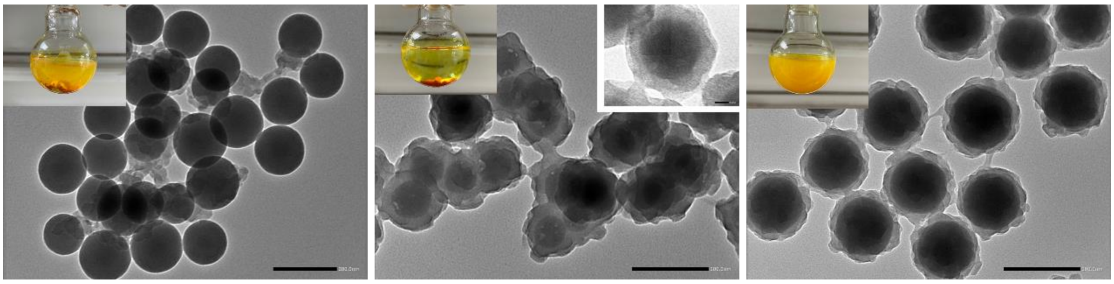

Supplementary Figure 32. TEM images of SiO_2@COF nanospheres prepared (left) in the absence of PEI and PVP, with the participation of (medium) PEI alone and (right) PVP alone. The inset photos are the corresponding dispersions after the reactions. Scale bar is all 200 nm. A representative image of three replicates from each group is shown. In the absence of PEI and PVP, the COF shell is not encapsulated on SiO_2 and only phase-separated flake-like COF is observed, implying that the interaction between COF monomers and SiO_2 nanospheres is very weak. When only PEI is modified on the SiO_2 surface, the core@shell structured SiO_2@COF nanocomposites with serious agglomeration is obtained, and the products are settled down at the bottom of the flask after the reaction, indicating the poor colloidal dispersity of the obtained samples. In

the case of PVP modified SiO_2, although the core@shell structured can be formed, the phase-separation is not avoided, and the thickness of the COF shell is obviously thinner than that of on the PEI-modified SiO_2.

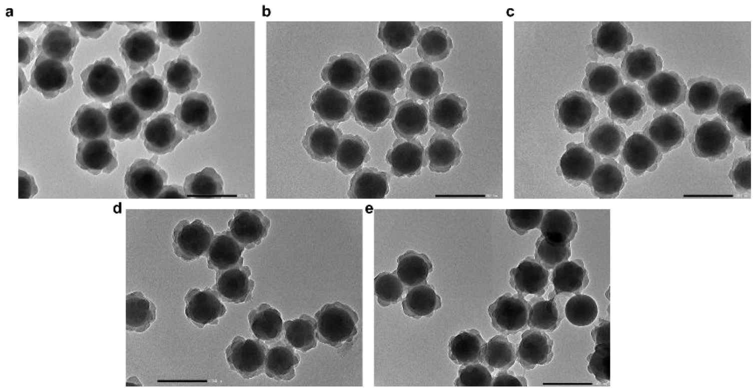

Supplementary Figure 33. TEM images of the obtained SiO_2@COF in the PVP solution at different concentration: (a) 2 mg/mL, (b) 4 mg/mL, (c) 10 mg/mL, (d) 20 mg/mL and (e) 40 mg/mL. Scale bar are 200 nm. A representative image of three replicates from each group is shown. With the absence of PEI, the COF domains on the SiO_2 nanospheres are "islands", which result in the irregular morphology of COF shell and even partially naked surface of the core. On the other hand, it is very difficult to tune the shell thickness by adjusting the PVP concentration.

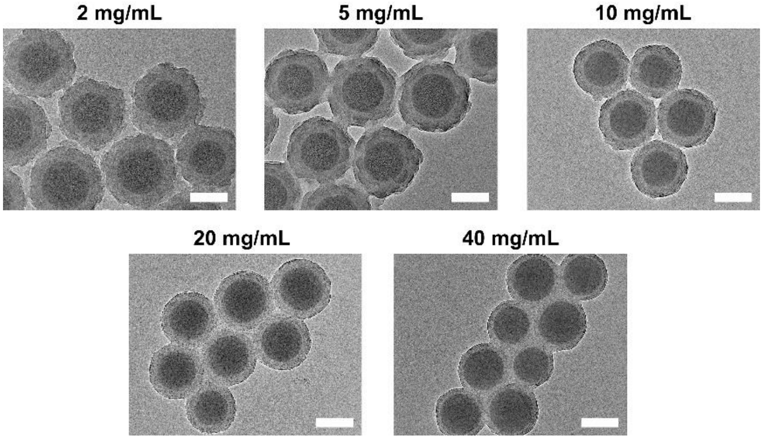

Supplementary Figure 34. TEM images of core@shell structured SiO_2@COF nanocomposites prepared by the dual-ligands assistant strategy at different PVP concentrations. Scale bars are 100 nm. A representative image of three replicates from each group is shown. It is clearly observed that the thickness of COF shell decreases

with the increased amount of PVP, further confirming that PVP can regulate the growth of COF shell. Cooperated with PEI, the growth of COF shell can be well manipulated to form monodispersed core@shell structure.

References

[1] T. C. Zhao, X. H. Zhu, C. T. Hung, P. Y. Wang, A. Elzatahry, A. A. Al-Khalaf, W. N. Hozzein, F. Zhang, X. M. Li, D. Y. Zhao, *J. Am. Chem. Soc.* **2018**, *140*, 10009-10015.

[2] H. Y. Liu, D. Chen, L. L. Li, T. L. Liu, L. F. Tan, X. L. Wu, F. Q. Tang, *Angew. Chem. Int. Edit* **2011**, *50*, 891-895.

[3] Z. J. Wang, N. Yu, X. Li, W. J. Yu, S. L. Han, X. L. Ren, S. W. Yin, M. Q. Li, Z. G. Chen, *Chem. Eng. J.* **2020**, *381*.

[4] W. Li, J. P. Yang, Z. X. Wu, J. X. Wang, B. Li, S. S. Feng, Y. H. Deng, F. Zhang, D. Y. Zhao, *J. Am. Chem. Soc.* **2012**, *134*, 11864-11867.

[5] X. M. Li, L. Zhou, Y. Wei, A. M. El-Toni, F. Zhang, D. Y. Zhao, *J. Am. Chem. Soc.* **2015**, *137*, 5903-5906.

[6] X. M. Li, R. Wang, F. Zhang, D. Y. Zhao, *Nano. Lett.* **2014**, *14*, 3634.

[7] X. M. Li, T. C. Zhao, Y. Lu, P. Y. Wang, A. M. El-Toni, F. Zhang, D. Y. Zhao, *Adv. Mater.* **2017**, *29*.

[8] M. Y. Zhao, B. H. Li, Y. F. Wu, H. S. He, X. Y. Zhu, H. X. Zhang, C. R. Dou, L. S. Feng, Y. Fan, F. Zhang, *Adv. Mater.* 2020, 32, 2001172
