# Supplementary material for: Imparting multi-functionality to covalent organic framework nanoparticles by the dual-ligand assistant encapsulation strategy
Source: Nat Commun. 2021 Jul 27;12:4556. doi: 10.1038/s41467-021-24838-7 (PMC8316466; doi:10.1038/s41467-021-24838-7)
Supplement: Supplementary file 3 — Reporting Summary [file 41467_2021_24838_MOESM3_ESM.pdf]

nature research

Corresponding author(s): Xiaomin Li

Last updated by author(s): Jul 8, 2021

Reporting Summary

Nature Research wishes to improve the reproducibility of the work that we publish. This form provides structure for consistency and transparency in reporting. For further information on Nature Research policies, see our Editorial Policies and the Editorial Policy Checklist.

Statistics

For all statistical analyses, confirm that the following items are present in the figure legend, table legend, main text, or Methods section.

n/a	Confirmed	
☐	☒	The exact sample size (n) for each experimental group/condition, given as a discrete number and unit of measurement
☐	☒	A statement on whether measurements were taken from distinct samples or whether the same sample was measured repeatedly
☐	☒	The statistical test(s) used AND whether they are one- or two-sided<br>*Only common tests should be described solely by name; describe more complex techniques in the Methods section.*
☒	☐	A description of all covariates tested
☒	☐	A description of any assumptions or corrections, such as tests of normality and adjustment for multiple comparisons
☐	☒	A full description of the statistical parameters including central tendency (e.g. means) or other basic estimates (e.g. regression coefficient) AND variation (e.g. standard deviation) or associated estimates of uncertainty (e.g. confidence intervals)
☐	☒	For null hypothesis testing, the test statistic (e.g. F, t, r) with confidence intervals, effect sizes, degrees of freedom and P value noted<br>*Give P values as exact values whenever suitable.*
☒	☐	For Bayesian analysis, information on the choice of priors and Markov chain Monte Carlo settings
☒	☐	For hierarchical and complex designs, identification of the appropriate level for tests and full reporting of outcomes
☒	☐	Estimates of effect sizes (e.g. Cohen's d, Pearson's r), indicating how they were calculated

Our web collection on statistics for biologists contains articles on many of the points above.

Software and code

Policy information about availability of computer code

Data collection	None
Data analysis	Image management: Adobe Photoshop CS6;<br>Data representation: OriginPro 8.5, Microsoft Powerpoint 2016, 3D Studio Max 2018, ChemBioDraw 8.0

For manuscripts utilizing custom algorithms or software that are central to the research but not yet described in published literature, software must be made available to editors and reviewers. We strongly encourage code deposition in a community repository (e.g. GitHub). See the Nature Research guidelines for submitting code & software for further information.

Data

Policy information about availability of data

All manuscripts must include a data availability statement. This statement should provide the following information, where applicable:

- Accession codes, unique identifiers, or web links for publicly available datasets
- A list of figures that have associated raw data
- A description of any restrictions on data availability

All relevant data supporting the findings of this study are included within the article and supplementary information files. Any other data are available from the authors upon reasonable request. The source data underlying Fig. 1g, 1h, 2g, 2h, 2i, 4d, 5b, 5d, 6c and Supplementary Fig. 6, 7, 22b, 24, 26, and 29 are provided as a Source Data file.

Field-specific reporting

Please select the one below that is the best fit for your research. If you are not sure, read the appropriate sections before making your selection.

- [x] Life sciences
- [ ] Behavioural & social sciences
- [ ] Ecological, evolutionary & environmental sciences

For a reference copy of the document with all sections, see nature.com/documents/nr-reporting-summary-flat.pdf

Life sciences study design

All studies must disclose on these points even when the disclosure is negative.

Sample size	Sample sizes were chosen based on the minimum number of animals/replicates to support meaningful conclusions, and the sample size (n) of each experiment is provided in the corresponding Figure legends in the paper and supplementary information.
Data exclusions	No data were excluded from the analyses.
Replication	Cell and animal experiments were replicated using samples from 3-5 independent biological samples. Exact numbers for each individual type of experiment are provided in Figure legends and in the Source Data file. All attempts at replicating results were successful.
Randomization	The cells were randomly seeded in a cell-culture plates and animals were randomly divided into different groups. Treatments were randomly allocated on each sample.
Blinding	Investigators were not blinded to group allocation during data collection and analysis due to limited operators and funds budget.

Reporting for specific materials, systems and methods

We require information from authors about some types of materials, experimental systems and methods used in many studies. Here, indicate whether each material, system or method listed is relevant to your study. If you are not sure if a list item applies to your research, read the appropriate section before selecting a response.

Materials & experimental systems

n/a	Involved in the study	
x		Antibodies
	x	Eukaryotic cell lines
x		Palaeontology and archaeology
	x	Animals and other organisms
x		Human research participants
x		Clinical data
x		Dual use research of concern

Methods

n/a	Involved in the study	
x		ChIP-seq
x		Flow cytometry
x		MRI-based neuroimaging

Eukaryotic cell lines

Policy information about cell lines

Cell line source(s)	Murine breast cancer cell line (4T1 cells) and human umbilical vein endothelial cell line (HUVECs) were purchased from cell bank of Chinese academy of science (Shanghai, China) and used in experiments.
Authentication	Cell lines were authenticated by Cell Bank/Stem Cell Bank, Chinese Academy of Sciences using specific methods (Karyotyping, DNA barcoding, PCR assays with species-specific primers, etc.)
Mycoplasma contamination	The cell lines were tested negative for mycoplasma contamination.
Commonly misidentified lines (See ICLAC register)	There are no misidentified cell lines in the experiment.

Animals and other organisms

Policy information about studies involving animals; ARRIVE guidelines recommended for reporting animal research

Laboratory animals	4~6 week-old female nude Balb/c mice were commercially supplied by Slac Laboratory Animal Co. Ltd. (Shanghai, China) and used in experiments. These mice were housed in ventilated stainless-steel cages under standard conditions (light: 12 h light/dark cycle, ambient temperature: 25 ± 2 °C, humidity: 60 ± 10%), which were fed with pellet food ad libitum and sterilized water.
Wild animals	The study do not involved wild animals.

April 2020

Field-collected samples	No field-collected samples were used in the study.
Ethics oversight	All the animal experiments were approved by the Shanghai Science and Technology Committee (SYXK2014-0029, Shanghai, China) and performed in agreement with the guidelines of the Department of Laboratory Animal Science, Fudan University.

Note that full information on the approval of the study protocol must also be provided in the manuscript.
